# 4-(4-Amino­phenyl­sulfon­yl)anilinium toluene-4-sulfonate

**DOI:** 10.1107/S1600536813033023

**Published:** 2013-12-14

**Authors:** Graham Smith, Urs D. Wermuth

**Affiliations:** a Science and Engineering Faculty, Queensland University of Technology, GPO Box 2434, Brisbane, Queensland 4001, Australia

## Abstract

In the title *p*-toluene­sulfonate salt of the drug dapsone, C_12_H_13_N_2_O_2_S^+^·C_7_H_7_O_3_S^−^, the dihedral angle between the two aromatic rings of the dapsone monocation is 70.19 (17)° and those between these rings and that of the *p*-toluene­sulfonate anion are 72.34 (17) and 46.43 (17)°. All amine and aminium H atoms are involved in inter­molecular N—H⋯O hydrogen-bonding associations with sulfonyl O-atom acceptors as well as one of the sulfone O atoms, giving a three-dimensional structure.

## Related literature   

For drug applications of dapsone, see: Wilson *et al.* (1991 ▶). For the structures of dapsone solvates, see: Kus’mina *et al.* (1981 ▶); Lemmer *et al.* (2012 ▶). For the structures of adducts and a salt of dapsone, see: Smith & Wermuth (2012*a* ▶,*b* ▶, 2013 ▶).
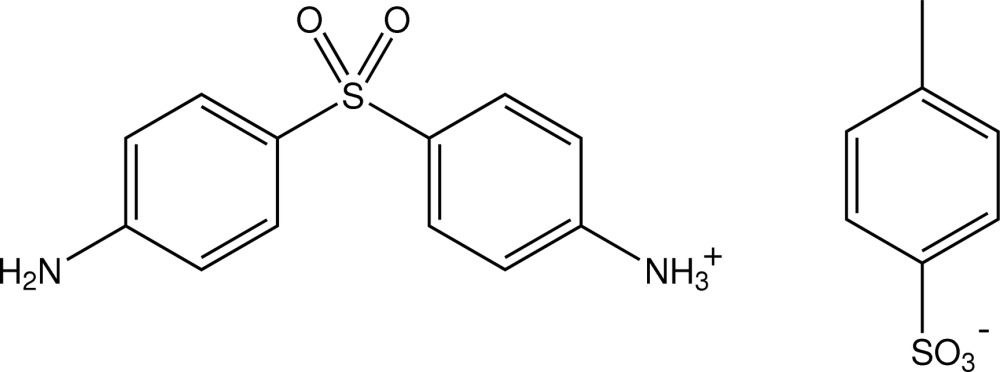


## Experimental   

### 

#### Crystal data  

C_12_H_13_N_2_O_2_S^+^·C_7_H_7_O_3_S^−^
*M* *_r_* = 420.49
Monoclinic, 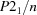

*a* = 5.9516 (9) Å
*b* = 25.147 (3) Å
*c* = 12.4506 (15) Å
β = 94.908 (11)°
*V* = 1856.6 (4) Å^3^
*Z* = 4
Mo *K*α radiation
μ = 0.32 mm^−1^
*T* = 200 K
0.25 × 0.12 × 0.12 mm

#### Data collection  

Oxford Diffraction Gemini-S CCD-detector diffractometer
Absorption correction: multi-scan (*CrysAlis PRO*; Agilent, 2013 ▶) *T*_min_ = 0.935, *T*_max_ = 0.980
6908 measured reflections
3650 independent reflections
2653 reflections with *I* > 2σ(*I*)
*R*_int_ = 0.046

#### Refinement  

*R*[*F* ^2^ > 2σ(*F* ^2^)] = 0.061
*wR*(*F* ^2^) = 0.161
*S* = 1.02
3650 reflections
253 parameters
H-atom parameters constrained
Δρ_max_ = 0.33 e Å^−3^
Δρ_min_ = −0.39 e Å^−3^

### 

Data collection: *CrysAlis PRO* (Agilent, 2013 ▶); cell refinement: *CrysAlis PRO*; data reduction: *CrysAlis PRO*; program(s) used to solve structure: *SIR92* (Altomare *et al.*, 1993 ▶); program(s) used to refine structure: *SHELXL97* (Sheldrick, 2008 ▶); molecular graphics: *PLATON* (Spek, 2009 ▶); software used to prepare material for publication: *PLATON*.

## Supplementary Material

Crystal structure: contains datablock(s) global, I. DOI: 10.1107/S1600536813033023/sj5377sup1.cif

Structure factors: contains datablock(s) I. DOI: 10.1107/S1600536813033023/sj5377Isup2.hkl

Supporting information file. DOI: 10.1107/S1600536813033023/sj5377Isup3.cml

Additional supporting information:  crystallographic information; 3D view; checkCIF report

## Figures and Tables

**Table 1:** Hydrogen-bond geometry (Å, °)

*D*—H⋯*A*	*D*—H	H⋯*A*	*D*⋯*A*	*D*—H⋯*A*
N4—H41⋯O13*A* ^i^	0.86	1.91	2.759 (4)	165
N4—H42⋯O11^ii^	0.83	2.24	3.008 (4)	153
N4—H43⋯O11*A* ^iii^	0.86	1.89	2.718 (4)	160
N41—H411⋯O12*A*	0.90	2.18	3.012 (4)	152
N41—H412⋯O13*A* ^iv^	0.97	2.46	3.369 (4)	155

Symmetry codes: (i) 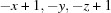; (ii) 
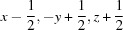
; (iii) 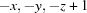; (iv) 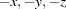.

**Fig. 1.**
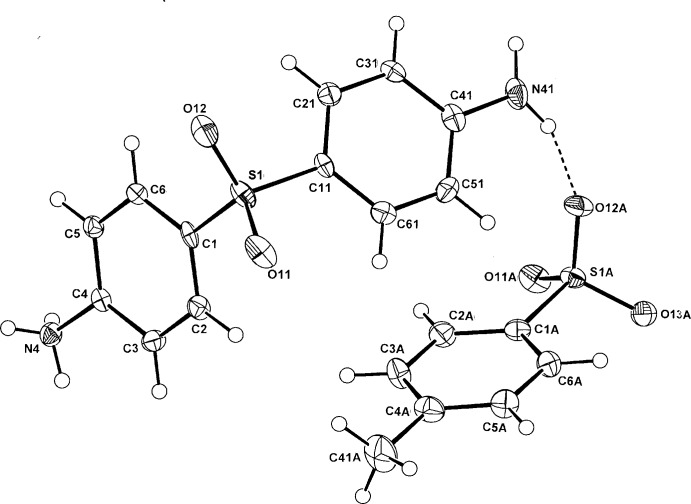
The molecular conformation and atom-numbering scheme for the dapsone monocation and p-toluenesulfonate anion in the title salt. Non-H atoms are shown as 40% probability displacement ellipsoids and the inter-species hydrogen bond is shown as a dashed line.

**Fig. 2.**
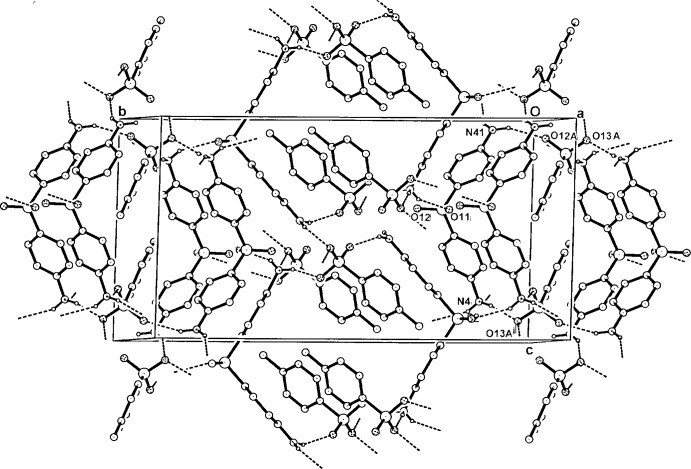
The hydrogen-bonding in the title salt, viewed down the a axial direction of the unit cell. Hydrogen bonds are shown as dashed lines. For symmetry codes see Table 1.

## supplementary crystallographic information

### 1. Comment

Dapsone [4-(4-aminophenylsulfonyl)aniline] is a very weak Lewis base which finds use as an anti-leprotic, anti-malarial and leprostatic drug (Wilson *et al.*, 1991). The structure of four dapsone solvates are known: the 0.33hydrate (Kus'mina *et al.*, 1981) and the (2:1) dichloromethane, (1:1) 1,4-dioxane and (1:1) tetrahydrofuran solvates (Lemmer *et al.*, 2012) but adducts or salts of this compound are not common. We have reported the structures of a (1:2) co-crystalline adduct with 1,3,5-trinitrobenzene (Smith & Wermuth, 2012*a*) and (1:1) adducts with 3,5-dinitrobenzoic acid (Smith & Wermuth, 2012*b*) and 5-nitroisophthalic acid (Smith & Wermuth, 2013) but only one proton-transfer salt structure is known, with 3,5-dinitrosalicylic acid (a monohydrate) (Smith & Wermuth, 2013). Reported herein is the structure of a second salt of dapsone, with *p*-toluenesulfonic acid, C_12_H_13_N_2_O_2_S^+^ C_7_H_7_O_3_S^-^.

In the structure of the title salt (Fig. 1), the conformation of the dapsone monocation as indicated by the inter-ring dihedral angle [70.19 (17)°], compares with 78.27 (9)° in the 3,5-dinitrosalicylic acid salt (Smith & Wermuth, 2013) and 75.4 (2)° in the 3,5-dinitobenzoic acid adduct (Smith & Wermuth, 2012*b*). The conformation of the title compound is influenced by short intramolecular ring *C*—*H*···O_sulfone_ interactions [C6—H···O12, 2.918 (4) Å and C21—H···O12, 2.925 (4) Å]. The angles between the *p*-toluenesulfonate ring and the aniline and anilinium rings respectively, are 46.43 (17) and 72.34 (17)°.

In the crystal, all amine and aminium H-atoms are involved in intermolecular N—H···O hydrogen-bonding associations with sulfonyl O-atom acceptors as well as with one of the sulfone O-atoms (O11) (Table 1). The resulting structure is a three-dimensional framework (Fig. 2). No π–π interactions are found between the cation and anion ring systems [minimum ring centroid separation = 4.534 (2) Å].

### 2. Experimental

The title compound was prepared by the reaction of 4-(4-aminophenylsulfonyl)aniline (dapsone) with *p*-toluenesulfonic acid by heating together for 15 min under reflux, 1 mmol quantities of the two reagents in 50 ml of 50% ethanol–water. Partial room-temperature evaporation of the solvent provided poorly-formed colourless crystal aggregates of the title salt from which a specimen was cleaved for the X-ray analysis.

### 3. Refinement

All H atoms potentially involved in hydrogen-bonding associations were located in a difference-Fourier analysis but were subsequently constrained, with *U*_iso_(H) = 1.2*U*_eq_(N). Other H-atoms were included at calculated positions [C—H = 0.95 Å (aromatic) or 0.98 Å (methyl)] and also treated as riding, with *U*_iso_(H) = 1.2 or 1.5*U*_eq_(C).

### Crystal data

**Table:**

C_12_H_13_N_2_O_2_S^+^·C_7_H_7_O_3_S^−^	*F*(000) = 880
*M**_r_* = 420.49	*D*_x_ = 1.504 Mg m^−^^3^
Monoclinic, *P*2_1_/*n*	Mo *K*α radiation, λ = 0.71073 Å
Hall symbol: -P 2yn	Cell parameters from 1570 reflections
*a* = 5.9516 (9) Å	θ = 3.6–27.2°
*b* = 25.147 (3) Å	µ = 0.32 mm^−^^1^
*c* = 12.4506 (15) Å	*T* = 200 K
β = 94.908 (11)°	Prism, colourless
*V* = 1856.6 (4) Å^3^	0.25 × 0.12 × 0.12 mm
*Z* = 4	

### Data collection

**Table:**

Oxford Diffraction Gemini-S CCD-detector diffractometer	3650 independent reflections
Radiation source: Enhance (Mo) X-ray source	2653 reflections with *I* > 2σ(*I*)
Graphite monochromator	*R*_int_ = 0.046
Detector resolution: 16.077 pixels mm^-1^	θ_max_ = 26.0°, θ_min_ = 3.2°
ω scans	*h* = −7→7
Absorption correction: multi-scan (*CrysAlis PRO*; Agilent, 2013)	*k* = −31→19
*T*_min_ = 0.935, *T*_max_ = 0.980	*l* = −7→15
6908 measured reflections	

### Refinement

**Table:**

Refinement on *F*^2^	Primary atom site location: structure-invariant direct methods
Least-squares matrix: full	Secondary atom site location: difference Fourier map
*R*[*F*^2^ > 2σ(*F*^2^)] = 0.061	Hydrogen site location: inferred from neighbouring sites
*wR*(*F*^2^) = 0.161	H-atom parameters constrained
*S* = 1.02	*w* = 1/[σ^2^(*F*_o_^2^) + (0.0672*P*)^2^ + 0.6392*P*] where *P* = (*F*_o_^2^ + 2*F*_c_^2^)/3
3650 reflections	(Δ/σ)_max_ < 0.001
253 parameters	Δρ_max_ = 0.33 e Å^−^^3^
0 restraints	Δρ_min_ = −0.39 e Å^−^^3^

### Special details

**Table:**

Geometry. Bond distances, angles *etc*. have been calculated using the rounded fractional coordinates. All su's are estimated from the variances of the (full) variance-covariance matrix. The cell e.s.d.'s are taken into account in the estimation of distances, angles and torsion angles
Refinement. Refinement of *F*^2^ against ALL reflections. The weighted *R*-factor *wR* and goodness of fit *S* are based on *F*^2^, conventional *R*-factors *R* are based on *F*, with *F* set to zero for negative *F*^2^. The threshold expression of *F*^2^ > σ(*F*^2^) is used only for calculating *R*-factors(gt) *etc*. and is not relevant to the choice of reflections for refinement. *R*-factors based on *F*^2^ are statistically about twice as large as those based on *F*, and *R*- factors based on ALL data will be even larger.

### Fractional atomic coordinates and isotropic or equivalent isotropic displacement parameters (Å^2^)

**Table:**

	*x*	*y*	*z*	*U*_iso_*/*U*_eq_	
S1	0.31318 (17)	0.24657 (3)	0.40362 (7)	0.0267 (3)	
O11	0.5538 (5)	0.25511 (10)	0.4069 (2)	0.0372 (9)	
O12	0.1671 (5)	0.29205 (9)	0.3968 (2)	0.0377 (9)	
N4	0.1704 (5)	0.13828 (12)	0.8172 (2)	0.0243 (9)	
N41	0.0383 (6)	0.10417 (12)	0.0404 (3)	0.0344 (10)	
C1	0.2645 (6)	0.21160 (12)	0.5225 (3)	0.0205 (10)	
C2	0.4324 (6)	0.17907 (14)	0.5693 (3)	0.0245 (11)	
C3	0.4005 (6)	0.15377 (14)	0.6663 (3)	0.0247 (11)	
C4	0.2020 (6)	0.16202 (12)	0.7128 (3)	0.0189 (10)	
C5	0.0323 (6)	0.19321 (13)	0.6645 (3)	0.0214 (10)	
C6	0.0637 (6)	0.21865 (13)	0.5689 (3)	0.0230 (11)	
C11	0.2346 (6)	0.20430 (13)	0.2959 (3)	0.0205 (10)	
C21	0.0160 (6)	0.20665 (14)	0.2470 (3)	0.0250 (11)	
C31	−0.0465 (6)	0.17356 (13)	0.1623 (3)	0.0241 (11)	
C41	0.1045 (7)	0.13691 (13)	0.1250 (3)	0.0254 (11)	
C51	0.3230 (6)	0.13441 (14)	0.1764 (3)	0.0275 (11)	
C61	0.3863 (6)	0.16748 (14)	0.2607 (3)	0.0261 (12)	
S1A	0.33152 (15)	−0.04463 (3)	0.14993 (8)	0.0244 (3)	
O11A	0.1942 (4)	−0.07383 (10)	0.2210 (2)	0.0339 (9)	
O12A	0.2028 (4)	−0.00796 (10)	0.0801 (2)	0.0331 (9)	
O13A	0.4701 (4)	−0.08006 (10)	0.0909 (2)	0.0314 (8)	
C1A	0.5187 (6)	−0.00617 (13)	0.2370 (3)	0.0238 (11)	
C2A	0.4538 (6)	0.00940 (15)	0.3363 (3)	0.0306 (12)	
C3A	0.5965 (7)	0.04085 (15)	0.4021 (3)	0.0329 (12)	
C4A	0.8047 (7)	0.05602 (15)	0.3713 (3)	0.0340 (12)	
C5A	0.8647 (6)	0.04020 (15)	0.2715 (3)	0.0315 (12)	
C6A	0.7228 (6)	0.00919 (14)	0.2039 (3)	0.0275 (11)	
C41A	0.9617 (8)	0.08898 (19)	0.4466 (4)	0.0532 (17)	
H2	0.56820	0.17400	0.53570	0.0290*	
H3	0.51380	0.13120	0.69970	0.0290*	
H5	−0.10570	0.19720	0.69680	0.0260*	
H6	−0.05120	0.24080	0.53540	0.0280*	
H21	−0.08940	0.23110	0.27220	0.0300*	
H31	−0.19530	0.17560	0.12830	0.0290*	
H41	0.29640	0.12250	0.83680	0.0290*	
H42	0.14260	0.16160	0.86140	0.0290*	
H43	0.06360	0.11530	0.82050	0.0290*	
H51	0.42800	0.10950	0.15240	0.0330*	
H61	0.53450	0.16530	0.29540	0.0320*	
H411	0.11190	0.07320	0.03200	0.0410*	
H412	−0.11720	0.10820	0.01020	0.0410*	
H2A	0.31240	−0.00140	0.35880	0.0370*	
H3A	0.55110	0.05230	0.46970	0.0400*	
H5A	1.00640	0.05080	0.24890	0.0380*	
H6A	0.76610	−0.00130	0.13530	0.0330*	
H41A	1.09020	0.10080	0.40850	0.0800*	
H42A	1.01600	0.06740	0.50910	0.0800*	
H43A	0.88040	0.12000	0.47090	0.0800*	

### Atomic displacement parameters (Å^2^)

**Table:**

	*U*^11^	*U*^22^	*U*^33^	*U*^12^	*U*^13^	*U*^23^
S1	0.0384 (6)	0.0232 (5)	0.0178 (5)	−0.0097 (4)	−0.0024 (4)	0.0024 (4)
O11	0.0397 (17)	0.0460 (17)	0.0256 (15)	−0.0258 (14)	0.0008 (13)	0.0054 (13)
O12	0.067 (2)	0.0179 (13)	0.0270 (15)	0.0014 (13)	−0.0020 (14)	0.0015 (12)
N4	0.0212 (16)	0.0297 (16)	0.0225 (16)	−0.0006 (13)	0.0051 (13)	−0.0015 (14)
N41	0.053 (2)	0.0245 (16)	0.0255 (17)	0.0009 (15)	0.0028 (16)	−0.0072 (14)
C1	0.033 (2)	0.0158 (16)	0.0127 (17)	−0.0093 (15)	0.0012 (15)	−0.0047 (14)
C2	0.0224 (19)	0.032 (2)	0.0196 (18)	−0.0037 (16)	0.0056 (16)	0.0030 (16)
C3	0.0213 (19)	0.0253 (18)	0.027 (2)	0.0029 (15)	−0.0001 (16)	−0.0011 (16)
C4	0.0245 (19)	0.0162 (16)	0.0162 (17)	−0.0054 (15)	0.0027 (15)	0.0006 (14)
C5	0.0185 (18)	0.0260 (18)	0.0195 (18)	−0.0032 (15)	0.0010 (15)	−0.0039 (16)
C6	0.025 (2)	0.0253 (18)	0.0181 (18)	0.0033 (16)	−0.0014 (16)	−0.0012 (16)
C11	0.0280 (19)	0.0205 (17)	0.0128 (16)	−0.0046 (15)	0.0015 (15)	0.0005 (15)
C21	0.030 (2)	0.0215 (17)	0.0234 (19)	0.0017 (16)	0.0011 (16)	0.0011 (16)
C31	0.026 (2)	0.0237 (18)	0.0213 (18)	−0.0031 (16)	−0.0048 (16)	0.0011 (16)
C41	0.039 (2)	0.0172 (17)	0.0200 (18)	−0.0057 (16)	0.0033 (17)	0.0031 (15)
C51	0.029 (2)	0.0234 (18)	0.031 (2)	0.0055 (16)	0.0073 (18)	−0.0014 (17)
C61	0.024 (2)	0.029 (2)	0.025 (2)	−0.0019 (16)	0.0003 (16)	0.0029 (17)
S1A	0.0234 (5)	0.0236 (5)	0.0251 (5)	−0.0014 (4)	−0.0042 (4)	0.0005 (4)
O11A	0.0294 (15)	0.0369 (15)	0.0349 (16)	−0.0134 (12)	−0.0001 (12)	0.0049 (13)
O12A	0.0332 (16)	0.0354 (15)	0.0290 (15)	0.0083 (12)	−0.0075 (12)	0.0059 (13)
O13A	0.0323 (15)	0.0293 (13)	0.0316 (15)	0.0037 (12)	−0.0027 (12)	−0.0059 (12)
C1A	0.0204 (19)	0.0224 (18)	0.028 (2)	−0.0016 (15)	−0.0006 (16)	0.0029 (16)
C2A	0.024 (2)	0.039 (2)	0.029 (2)	−0.0027 (18)	0.0030 (17)	0.0007 (19)
C3A	0.038 (2)	0.039 (2)	0.022 (2)	−0.0068 (19)	0.0050 (18)	−0.0029 (18)
C4A	0.036 (2)	0.026 (2)	0.038 (2)	−0.0029 (18)	−0.0075 (19)	−0.0020 (19)
C5A	0.025 (2)	0.033 (2)	0.037 (2)	−0.0086 (17)	0.0053 (18)	−0.0017 (19)
C6A	0.030 (2)	0.0265 (19)	0.026 (2)	−0.0020 (17)	0.0023 (17)	−0.0014 (17)
C41A	0.053 (3)	0.052 (3)	0.053 (3)	−0.018 (2)	−0.005 (3)	−0.020 (3)

### Geometric parameters (Å, º)

**Table:**

S1—O11	1.445 (3)	C41—C51	1.401 (5)
S1—O12	1.435 (3)	C51—C61	1.367 (5)
S1—C1	1.767 (4)	C2—H2	0.9500
S1—C11	1.744 (4)	C3—H3	0.9500
S1A—C1A	1.773 (4)	C5—H5	0.9500
S1A—O13A	1.455 (3)	C6—H6	0.9500
S1A—O11A	1.454 (3)	C21—H21	0.9500
S1A—O12A	1.442 (3)	C31—H31	0.9500
N4—C4	1.457 (4)	C51—H51	0.9500
N41—C41	1.368 (5)	C61—H61	0.9500
N4—H42	0.8300	C1A—C2A	1.383 (5)
N4—H43	0.8600	C1A—C6A	1.371 (5)
N4—H41	0.8600	C2A—C3A	1.378 (5)
N41—H411	0.9000	C3A—C4A	1.382 (6)
N41—H412	0.9700	C4A—C5A	1.381 (5)
C1—C6	1.383 (5)	C4A—C41A	1.513 (6)
C1—C2	1.381 (5)	C5A—C6A	1.381 (5)
C2—C3	1.392 (5)	C2A—H2A	0.9500
C3—C4	1.375 (5)	C3A—H3A	0.9500
C4—C5	1.376 (5)	C5A—H5A	0.9500
C5—C6	1.378 (5)	C6A—H6A	0.9500
C11—C61	1.390 (5)	C41A—H41A	0.9800
C11—C21	1.390 (5)	C41A—H42A	0.9800
C21—C31	1.370 (5)	C41A—H43A	0.9800
C31—C41	1.394 (5)		
			
O11—S1—O12	118.50 (16)	C1—C2—H2	120.00
O11—S1—C1	106.46 (16)	C3—C2—H2	120.00
O11—S1—C11	108.20 (16)	C2—C3—H3	121.00
O12—S1—C1	107.72 (16)	C4—C3—H3	121.00
O12—S1—C11	108.61 (16)	C6—C5—H5	120.00
C1—S1—C11	106.77 (16)	C4—C5—H5	120.00
O11A—S1A—O13A	111.75 (15)	C1—C6—H6	120.00
O11A—S1A—C1A	105.06 (16)	C5—C6—H6	120.00
O12A—S1A—O13A	112.45 (15)	C11—C21—H21	120.00
O12A—S1A—C1A	107.06 (15)	C31—C21—H21	120.00
O13A—S1A—C1A	106.80 (16)	C41—C31—H31	119.00
O11A—S1A—O12A	113.12 (15)	C21—C31—H31	119.00
C4—N4—H41	105.00	C41—C51—H51	120.00
C4—N4—H42	110.00	C61—C51—H51	120.00
H41—N4—H42	111.00	C51—C61—H61	120.00
H41—N4—H43	108.00	C11—C61—H61	120.00
H42—N4—H43	105.00	S1A—C1A—C2A	119.5 (3)
C4—N4—H43	118.00	S1A—C1A—C6A	119.8 (3)
C41—N41—H412	116.00	C2A—C1A—C6A	120.8 (3)
H411—N41—H412	120.00	C1A—C2A—C3A	119.3 (3)
C41—N41—H411	120.00	C2A—C3A—C4A	121.0 (3)
S1—C1—C2	118.9 (3)	C3A—C4A—C5A	118.5 (4)
S1—C1—C6	119.7 (3)	C3A—C4A—C41A	120.0 (4)
C2—C1—C6	121.3 (3)	C5A—C4A—C41A	121.5 (4)
C1—C2—C3	119.3 (3)	C4A—C5A—C6A	121.3 (3)
C2—C3—C4	118.8 (3)	C1A—C6A—C5A	119.2 (3)
N4—C4—C3	119.7 (3)	C1A—C2A—H2A	120.00
N4—C4—C5	118.5 (3)	C3A—C2A—H2A	120.00
C3—C4—C5	121.8 (3)	C2A—C3A—H3A	120.00
C4—C5—C6	119.6 (3)	C4A—C3A—H3A	119.00
C1—C6—C5	119.2 (3)	C4A—C5A—H5A	119.00
S1—C11—C21	119.4 (3)	C6A—C5A—H5A	119.00
S1—C11—C61	120.7 (3)	C1A—C6A—H6A	120.00
C21—C11—C61	120.0 (3)	C5A—C6A—H6A	120.00
C11—C21—C31	119.6 (3)	C4A—C41A—H41A	109.00
C21—C31—C41	121.2 (3)	C4A—C41A—H42A	109.00
N41—C41—C51	121.3 (3)	C4A—C41A—H43A	109.00
N41—C41—C31	120.2 (4)	H41A—C41A—H42A	110.00
C31—C41—C51	118.5 (3)	H41A—C41A—H43A	110.00
C41—C51—C61	120.5 (3)	H42A—C41A—H43A	109.00
C11—C61—C51	120.2 (3)		
			
O11—S1—C1—C2	−28.1 (3)	C2—C3—C4—N4	−176.6 (3)
O11—S1—C1—C6	149.6 (3)	N4—C4—C5—C6	176.2 (3)
O12—S1—C1—C2	−156.2 (3)	C3—C4—C5—C6	−2.5 (5)
O12—S1—C1—C6	21.5 (3)	C4—C5—C6—C1	1.0 (5)
C11—S1—C1—C2	87.3 (3)	S1—C11—C61—C51	179.8 (3)
C11—S1—C1—C6	−95.0 (3)	C61—C11—C21—C31	−1.8 (5)
O11—S1—C11—C21	−153.5 (3)	S1—C11—C21—C31	180.0 (3)
O11—S1—C11—C61	28.2 (3)	C21—C11—C61—C51	1.6 (5)
O12—S1—C11—C21	−23.7 (3)	C11—C21—C31—C41	0.9 (5)
O12—S1—C11—C61	158.1 (3)	C21—C31—C41—N41	179.9 (3)
C1—S1—C11—C21	92.2 (3)	C21—C31—C41—C51	0.2 (5)
C1—S1—C11—C61	−86.0 (3)	C31—C41—C51—C61	−0.4 (5)
O12A—S1A—C1A—C2A	92.7 (3)	N41—C41—C51—C61	179.9 (3)
O12A—S1A—C1A—C6A	−85.3 (3)	C41—C51—C61—C11	−0.5 (5)
O13A—S1A—C1A—C2A	−146.7 (3)	S1A—C1A—C2A—C3A	−177.7 (3)
O13A—S1A—C1A—C6A	35.4 (3)	C6A—C1A—C2A—C3A	0.2 (5)
O11A—S1A—C1A—C2A	−27.8 (3)	S1A—C1A—C6A—C5A	178.5 (3)
O11A—S1A—C1A—C6A	154.2 (3)	C2A—C1A—C6A—C5A	0.5 (5)
S1—C1—C2—C3	176.3 (3)	C1A—C2A—C3A—C4A	−1.4 (6)
C2—C1—C6—C5	0.9 (5)	C2A—C3A—C4A—C5A	1.8 (6)
C6—C1—C2—C3	−1.4 (5)	C2A—C3A—C4A—C41A	−177.9 (4)
S1—C1—C6—C5	−176.8 (3)	C3A—C4A—C5A—C6A	−1.0 (6)
C1—C2—C3—C4	−0.1 (5)	C41A—C4A—C5A—C6A	178.7 (4)
C2—C3—C4—C5	2.0 (5)	C4A—C5A—C6A—C1A	−0.1 (6)

### Hydrogen-bond geometry (Å, º)

**Table:**

*D*—H···*A*	*D*—H	H···*A*	*D*···*A*	*D*—H···*A*
N4—H41···O13*A*^i^	0.86	1.91	2.759 (4)	165
N4—H42···O11^ii^	0.83	2.24	3.008 (4)	153
N4—H43···O11*A*^iii^	0.86	1.89	2.718 (4)	160
N41—H411···O12*A*	0.90	2.18	3.012 (4)	152
N41—H412···O13*A*^iv^	0.97	2.46	3.369 (4)	155
C2—H2···O11	0.95	2.59	2.918 (4)	101
C2*A*—H2*A*···O11*A*	0.95	2.56	2.907 (4)	102
C6—H6···O12	0.95	2.59	2.933 (4)	102
C21—H21···O12	0.95	2.58	2.935 (4)	102

Symmetry codes: (i) −*x*+1, −*y*, −*z*+1; (ii) *x*−1/2, −*y*+1/2, *z*+1/2; (iii) −*x*, −*y*, −*z*+1; (iv) −*x*, −*y*, −*z*.
